# *Acorus calamus* L. Essential Oil Induces Oxidative Stress and DNA Replication Disruptions in Root Meristem Cells of Two Fabaceae and Two Brassicaceae Species

**DOI:** 10.3390/ijms26104715

**Published:** 2025-05-14

**Authors:** Mateusz Wróblewski, Konrad Krajewski, Natalia Gocek, Aneta Żabka, Justyna T. Polit

**Affiliations:** 1 Department of Cytophysiology, Faculty of Biology and Environmental Protection, University of Lodz, 90-236 Lodz, Poland; konrad.s.krajewski@gmail.com (K.K.); natalia.gocek@biol.uni.lodz.pl (N.G.); aneta.zabka@biol.uni.lodz.pl (A.Ż.); justyna.polit@biol.uni.lodz.pl (J.T.P.); 2 Doctoral School of Exact and Natural Sciences, University of Lodz, 90-237 Lodz, Poland

**Keywords:** bioactive plant compounds, bioherbicide, cell cycle, DNA-strand breaks, γ-H2AX phosphorylation, TUNEL assay

## Abstract

Environmental concerns regarding synthetic herbicides have sparked interest in plant-derived bioactive compounds as eco-friendly alternatives. This study investigated the cellular targets of sweet flag essential oil (*Acorus calamus* L., SEO at IC_50_ concentration) in root meristem cells of Fabaceae (*Vicia faba*, *Lupinus luteus*) and Brassicaceae (*Brassica napus*, *Arabidopsis thaliana*), focusing on reactive oxygen species (ROS) accumulation (DAB, NBT staining), DNA replication dynamics (EdU labeling), and genome integrity (γ-H2AX immunocytochemistry, TUNEL assay, and DNA electrophoresis). SEO induced oxidative stress (200–250% of control depending on the species) and replication stress, causing DNA double-strand breaks in 50% of proliferating cells, confirmed by γ-H2AX/TUNEL. Consequently, cells were prolonged in the G_1_ phase, replication activity dropped to 70% of control in Fabaceae and 80% in Brassicaceae, and EdU incorporation intensity decreased to 80% and 70% of control, respectively. An increased proportion of cells replicating heterochromatin indicated slowed S-phase progression. Despite genotoxic effects, SEO did not trigger endoreplication, apoptotic DNA fragmentation, or extensive cell death. All species exhibited a uniform stress response, although sensitivity varied, which previously enabled the establishment of selective SEO doses between Fabaceae and Brassicaceae. These findings suggest that SEO exerts phytotoxicity by disrupting S-phase progression, supporting its potential as a selective bioherbicide.

## 1. Introduction

Environmental pollution caused by chemical pesticides is related to the intensification of agriculture, which aims to produce abundant and high-quality crop yields. Irrational use of pesticides contributes to their loss of function, increasing pest resistance, and, consequently, exceeding the determined doses and withdrawal of the plant protection product. The chemicals used are often accumulated in soil and crops, endangering the health of consumers [1,2,3]. These problems drive to search for efficient, biodegradable, natural compounds that neither accumulate in soil nor contaminate water and show no toxicity towards people and animals. Plant secondary metabolism products, including a huge variety of essential oils, seem to be a promising solution to the abovementioned problems [4]. The selective and diversified impact of essential oils on the germination and seedling development of several species brings attention to their potential use as biostimulators or bioherbicides [5]. A particularly important target for the action of bioactive compounds, including essential oils, is the cell cycle, as it is highly sensitive to environmental and chemical stress factors [6,7,8]. Moreover, proliferation, starting from the stage of germination and the formation of the primary root, directly determines plant growth. Inhibiting the cell cycle is therefore an effective strategy for eliminating unwanted weeds. Bioactive compounds can affect the cell cycle either directly or indirectly by inducing excessive production of reactive oxygen species (ROS), leading to oxidative stress [9]. This shift in balance toward harmful oxidation reactions results in damage to the structure and function of DNA, proteins, and lipids [10]. ROS pose a threat, not only to the structure of genetic material and the mitotic apparatus, but also to the function of molecular regulatory factors that form the basis of the action of cell cycle checkpoints, located at critical transitions between the G1/S and G2/M phases, as well as during key processes such as DNA replication and mitotic chromosome segregation [11].

The key regulators of the plant cell cycle are complexes of cyclin-dependent kinases (CDKs: CDKA, CDKB1, and CDKB2) with cyclins (Cyc A, B, D, and E). CDKs differ in their expression timing during the cell cycle and their functional specificity [12]. CDKA is present throughout the entire cycle, whereas CDKB1 and CDKB2 are primarily expressed from the S to M phase [13]. Cyclins are regulatory proteins whose concentrations fluctuate depending on the cell cycle phase. The activity of CDK-cyclin complexes is regulated both through phosphorylation/dephosphorylation processes and by interaction with CDK inhibitors (CKIs) belonging to the Kip-Related Protein (KRP) family [14]. In response to external signals, such as hormones and nutrient availability, cyclin D synthesis is induced. By binding to CDKA, cyclin D promotes the expression of cyclin E, another CDKA partner. Active CDKA-CycA and CDKA-CycE complexes phosphorylate, the Rb protein, leading to the release of the transcription factor E2F, which activates genes involved in DNA replication [15]. This marks the transition of the cell into the S phase, during which a sophisticated and tightly regulated machinery ensures DNA synthesis, allowing the parent cell to transmit an exact genetic copy to the two daughter cells. This process occurs simultaneously at multiple sites within the genome and is organized through the existence of replicons—replicative units that include the origin of replication, replication forks that branch out in opposite directions, and the replication termination sites. The origin sites are gradually activated during the S phase. Replicons can be classified as early or late, depending on when they are activated within the S phase. Early replicons are initiated at the beginning of the S phase and typically encompass actively transcribed euchromatin regions, while late replicons replicate heterochromatic areas of the genome at later stages. In the classical cell cycle, each replicon, regardless of activation timing, is only replicated once during each S phase [16].

DNA synthesis requires the coordinated action of multiple enzymes and stabilizing proteins. Helicase unwinds the DNA by breaking hydrogen bonds, allowing access to the template strand. Single-stranded DNA binding proteins (SSBs) prevent re-annealing and degradation, stabilizing the unwound DNA. Topoisomerase, ahead of the replication fork, alleviates supercoiling by cutting and rejoining DNA strands. Primase synthesizes short RNA primers, initiating replication for DNA polymerases. DNA polymerase α facilitates initiation, while δ and ε elongate and proofread the lagging and leading strands, respectively, adding nucleotides in a 5′ → 3′ direction. Exonucleases remove RNA primers and excise replication errors. DNA ligase seals Okazaki fragments, ensuring strand continuity [6].

To maintain genome stability, the complex process of genetic material duplication is tightly regulated by a sophisticated system of molecular mechanisms that enable cells to detect, signal, and repair DNA damage in a process known as DNA Damage Response (DDR) [12]. The detection and identification of DNA damage are carried out by specialized sensors: the MRN complex (MRE11-RAD50-NBS1), which detects double-strand breaks (DSBs) and recruits the ATM kinase (Ataxia-Telangiectasia Mutated); RPA (Replication Protein A), a protein that recognizes single-stranded DNA (ssDNA) and stabilizes the replication fork; and the 9-1-1 complex (RAD9-RAD1-HUS1), which functions as a ring that encircles DNA at single- to double-strand junctions and activates ATR kinases (Ataxia Telangiectasia Mutated and Rad3-related). Activated by both single- and double-strand DNA breaks, as well as by replication fork slowing or stalling, ATM and ATR kinases initiate the response. This results in the phosphorylation of histone H2A.X, leading to the recruitment of repair proteins to the damage sites and activation of the repair pathway [12,17]. Phosphorylation also occurs in cell cycle regulatory proteins, including the transcription factor protein SOG1 (Suppressor of Gamma Response 1), a functional equivalent of the p53 protein in animal cells but with no sequence homology to it [18], which induces the expression of cyclin inhibitors and genes associated with DNA repair or programmed cell death if the damage is too severe. Additionally, the WEE1 kinase is phosphorylated, which inhibits the activity of CDK/Cyclin complexes, thus halting the cell cycle at the S or G2/M phase and allowing time for the activation of repair mechanisms and DNA repair [12]. If the damage is too extensive to repair, DDR can trigger programmed cell death (PCD, apoptosis-like death) or induce cell senescence. Regulation of the S phase in plant cells combines mechanisms characteristic of other eukaryotes with unique adaptations that enable plant cells to respond to changing environmental conditions, such as nutrient availability and abiotic stress. This highlights the plasticity of the cell cycle in plants. Under unfavorable conditions, plants can slow down or even halt replication to protect cells from duplicating damaged DNA [17].

This study builds on previous research on SEO composition and its biological activity by examining its impact on plant root growth. The major compounds identified in SEO include phenylpropanoids (β-, α-, and γ-asarone), monoterpenes (α- and β-pinene, camphene, myrcene, and limonene), sesquiterpenes (cadinene, acorone, acorenone, and calamine), tannins, choline, and fatty acids [19]. Despite extensive studies on SEO, its specific effects on plant development and cellular responses remain insufficiently understood. This study examined the primary roots of two Fabaceae species (*Vicia faba* var. *minor* L. and *Lupinus luteus* L.) and two Brassicaceae species (*Brassica napus* L. and *Arabidopsis thaliana* L.) which were incubated for 24 h in sweet flag (*Acorus calamus* L.) essential oil (SEO) at an IC_50_ concentration—individually determined for each species—that inhibited root growth by 50% [20]. To test our hypothesis that *Acorus calamus* L. essential oil (SEO) inhibits root growth by inducing oxidative stress that disrupts DNA replication during the S phase of the cell cycle, we analyzed reactive oxygen species (ROS) accumulation, assessed DNA replication dynamics, and examined DNA integrity in root meristems. We further hypothesized that these disruptions might differ between species, revealing either species-specific responses, similarities within plant families, or broader distinctions between Fabaceae and Brassicaceae.

## 2. Results

### 2.1. The Impact of SEO on H_2_O_2_ and O_2_^•−^ in Root Meristematic Cells

**Figure 1:**
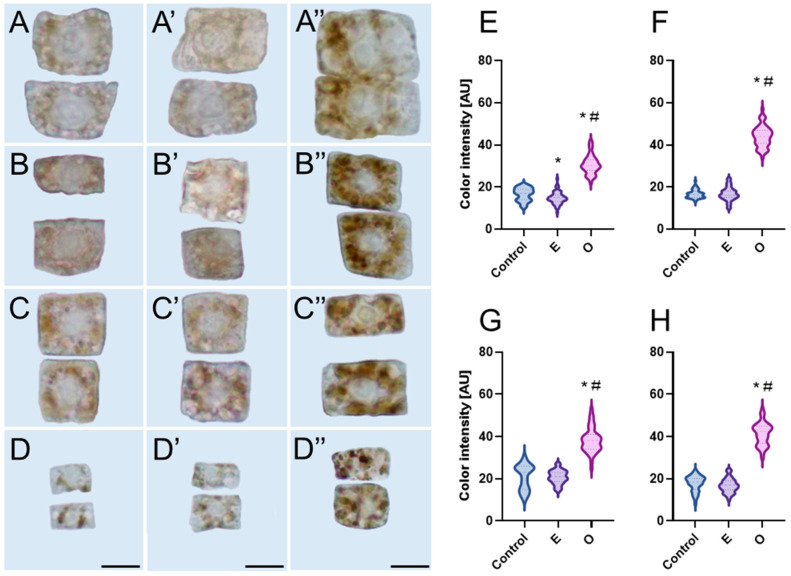
Generation of H_2_O_2_ visualized as brown deposits of oxidized diaminobenzidine (DAB) and corresponding cytometric evaluation of mean DAB staining intensity [arbitrary units, AU] in *Vicia faba* (**A**–**A”**,**E**), *Lupinus luteus* (**B**–**B”**,**F**), *Brassica napus* (**C**–**C”**,**G**), and *Arabidopsis thaliana* (**D**–**D”**,**H**) root meristem cells (N = 50) after 24h seedling incubation in water—Control (**A**–**D**), the emulsifier solution—E (**A’**–**D’**), or emulsified SEO at the IC_50_ concentration—O (**A”**–**D”**). Scale bar: 10 µm. The width of each violin plot represents the distribution of values along the y-axis; medians are indicated by dashed lines and quartiles by dotted lines. Statistical differences were assessed using Welch’s *t*-test and the Mann–Whitney test at *p* ≤ 0.05. Asterisk (*) and hash (#) marks denote significant differences compared to the control and the emulsifier, respectively.

One of the most commonly used methods to visualize the location of hydrogen peroxide (H_2_O_2_) is based on the oxidation of diaminobenzidine (DAB) by H_2_O_2_ produced in cells. The oxidized DAB appears as brown precipitates visible under a light microscope (Figure 1A–D”). In the roots of seedlings from all four studied species—previously incubated for 24 h in water (control), emulsifier, and emulsified SEO at an IC_50_ concentration—meristems treated with the emulsifier exhibited a low level of staining, similar to that of the control (Figure 1A–D’,E–H). In contrast, the most intense staining was observed in the root meristem zones treated with SEO, particularly in *L. luteus* and *A. thaliana*, where the staining reached approximately 250% of the control level (Figure 1B”,D”,F,H), and to a slightly lesser extent in *V. faba* and *B. napus*, where it was around 200% of the control (Figure 1A”,C”,E,G). Microscopic analyses revealed both the dispersed cytoplasmic location of DAB precipitates, as well as brownish deposits clearly grouped in oval structures primarily surrounding the cell nucleus, which always remained brighter than the other areas of the cell (Figure 1A”–D”).

**Figure 2:**
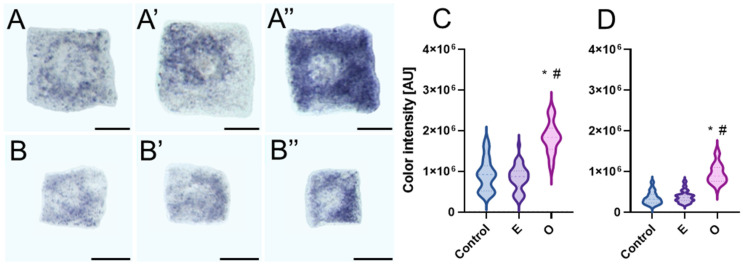
Generation of O_2_^•**−**^ detected by NBT staining and corresponding cytometric evaluation of mean NBT staining intensity [arbitrary units- AU] in *V. faba* (**A**–**A”**,**C**) and *B. napus* (**B**–**B”**,**D**) root meristem cells (N = 50) after 24 h of seedling incubation in water—Control (**A**,**B**), the emulsifier solution—E (**A’**,**B’**), or emulsified SEO at the IC_50_ concentration—O (**A”**,**B”**)**.** Scale bar: 10 µm. The width of each violin plot represents the distribution of values along the y-axis; medians are indicated by dashed lines and quartiles by dotted lines. Statistical differences were assessed using the Mann–Whitney test at *p* ≤ 0.05. Asterisk (*) and hash (#) marks denote significant differences compared to the control and the emulsifier, respectively.

The level of the second type of ROS analyzed, the superoxide anion radical (O_2_^•−^), was assessed using nitroblue tetrazolium (NBT) reduction. The yellow NBT solution enters the cells, where it is reduced in the presence of O_2_^•−^ to a blue, water-insoluble formazan, visible under a light microscope as precipitates (Figure 2A–B”). The reaction was conducted only on representatives of the Fabaceae and Brassicaceae families (*V. faba* and *B. napus*) in which a slightly lower H_2_O_2_ content was recorded. Similar to the identification of H_2_O_2_, a significant amount of O_2_^•−^ was detected in the root meristematic zone treated with SEO, compared to the control and emulsifier-treated meristems. In the microscopic images, dark blue formazan deposits formed fine granules scattered throughout the cytoplasm and concentrated near the cell nuclei (Figure 2A–B”). Despite the similarities in H_2_O_2_ content in the root meristematic cells of both species treated with SEO, the quantitative analysis of formazan deposits in individual cells, based on staining intensity, indirectly indicated a significantly higher level of O_2_^•−^ in the root meristems of *V. faba* compared to *B. napus*, while the assessment of staining intensity increase in SEO-treated cells relative to the control revealed an O_2_^•−^ content rise to 200% in *V. faba* and up to 275% in *B. napus* (Figure 2C,D).

### 2.2. Assessment of the Replication Process and the Percentage Distribution of Meristematic Cell Populations Based on DNA Content

The reduction of root growth to half of the control value under the influence of SEO, accompanied by an elevated ROS level in the meristematic zone, may result from disturbances in the cell cycle, where DNA replication during the S phase is a critical yet highly oxidative stress-sensitive stage. The first signs of these irregularities appear as disruptions in the distribution of cells across different interphase stages.

**Figure 3:**
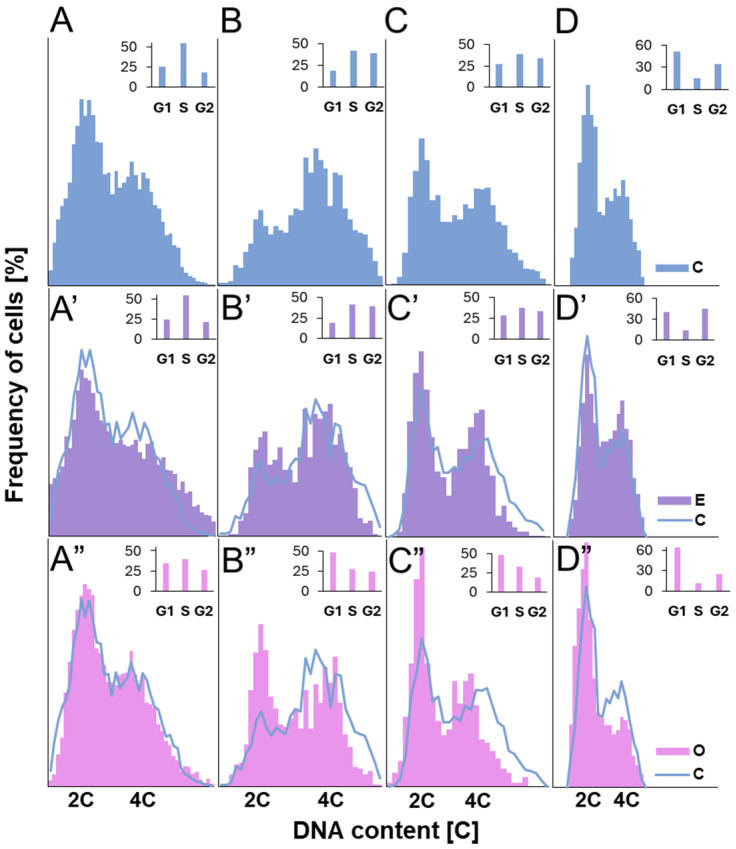
Frequency distribution [%] of nuclear DNA content [C] in root apical meristem cells of *V. faba* (**A**–**A”**), *L. luteus* (**B**–**B”**), *B. napus* (**C**–**C”**), and *A. thaliana* (**D**–**D”**) after 24 h seedling incubation in water—C (**A**–**D**), the emulsifier solution—E (**A’**–**D’**), or emulsified SEO at the IC_50_ concentration—O (**A”**–**D”**). Bar graphs on the right of each histogram show the percentages of cells in the G1, S, and G2 phases for the respective experimental series.

**Figure 4:**
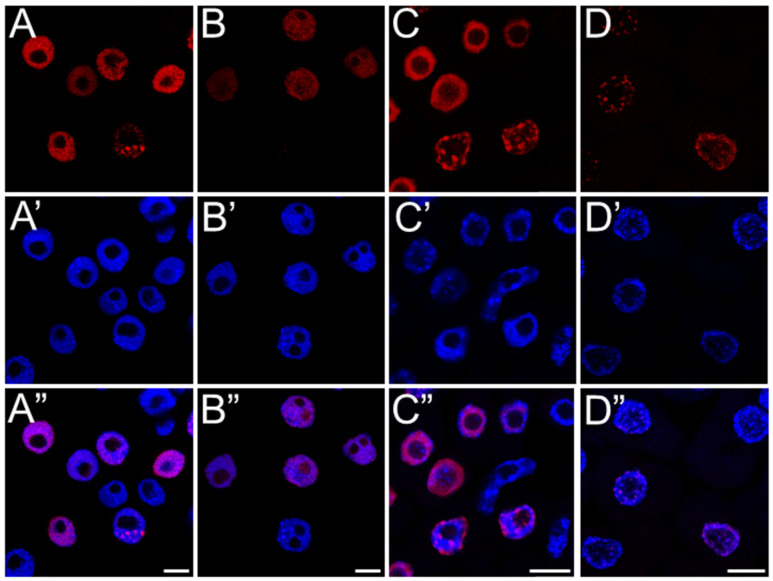
DNA replication detected by EdU assay in root meristem cells of *V. faba* (**A**,**B**) and *B. napus* (**C**,**D**) after 24 h seedling incubation in water (Control; (**A**,**C**)), or emulsified SEO at the IC_50_ concentration (**B**,**D**). Corresponding DNA staining with DAPI is shown in (**A’**–**D’**), and merged images in (**A”**–**D”**). Scale bar: 10 µm.

**Figure 5:**
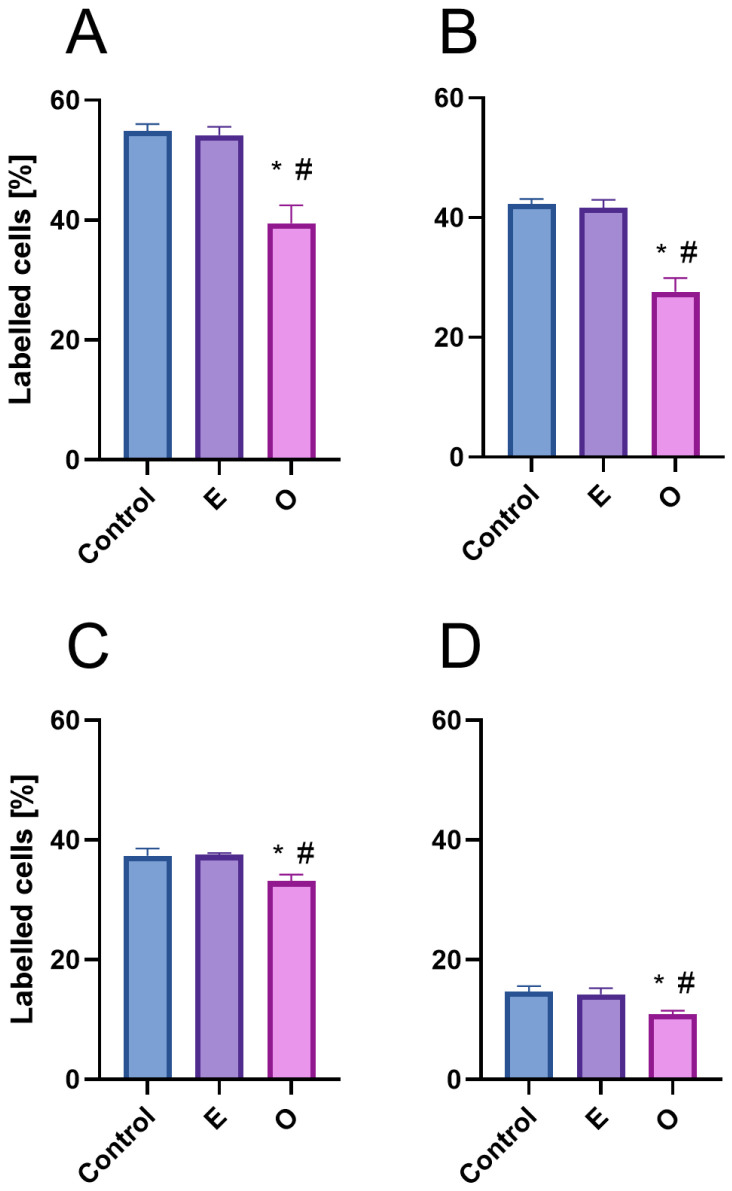
Labelling indices of EdU-positive root meristem cells of *V. faba* (**A**), *L. luteus* (**B**), *B. napus* (**C**) and *A. thaliana* (**D**) after 24 h seedling incubation in water—Control, the emulsifier solution—E, or emulsified SEO at the IC_50_ concentration—O. Data are presented as mean indices ± SEM from three biological replicates. Statistical differences were assessed using Student’s *t*-test at *p* ≤ 0.05. Asterisk (*) and hash (#) marks denote significant differences compared to the control and the emulsifier, respectively.

The primary methods for identifying cells in the G1, S, and G2 phases include cytophotometric assessment of DNA content in Feulgen-stained nuclei (Figure 3) and fluorescent detection of 5-ethynyl-2′-deoxyuridine (EdU), a thymidine analog that incorporates into newly synthesized DNA strands, thereby labeling the nuclei of replicating cells (Figure 4), and thus allowing the calculation of their percentage (labeling index, Figure 5).

The analysis revealed slight variations in population distributions among control meristems of different species and no significant changes in these distributions under the influence of the emulsifier (see Figure 3A–D’ and Figure 5). The distributions were characterized by the highest proportion of cells in the S phase in *V. faba* (approximately 50%), slightly lower but similar proportions in *L. luteus* and *B. napus* (around 40%), and the lowest percentage in *A. thaliana* (15%).

Exposure to SEO at the IC_50_ concentration caused a significant redistribution of meristematic cells across different interphase stages (Figure 3A”–D” and Figure 5). A pronounced increase in the number of cells with 2C DNA content, characteristic of the G1 phase, was observed, accompanied by a decrease in the number of cells with 4C DNA content in the G2 phase, as well as a reduction in the population of cells with intermediate DNA content (between 2C and 4C), characteristic of the S phase. Replicating cells accounted for nearly 70% of the control level in both Fabaceae species and approximately 80% in Brassicaceae species (Figure 5).

**Figure 6:**
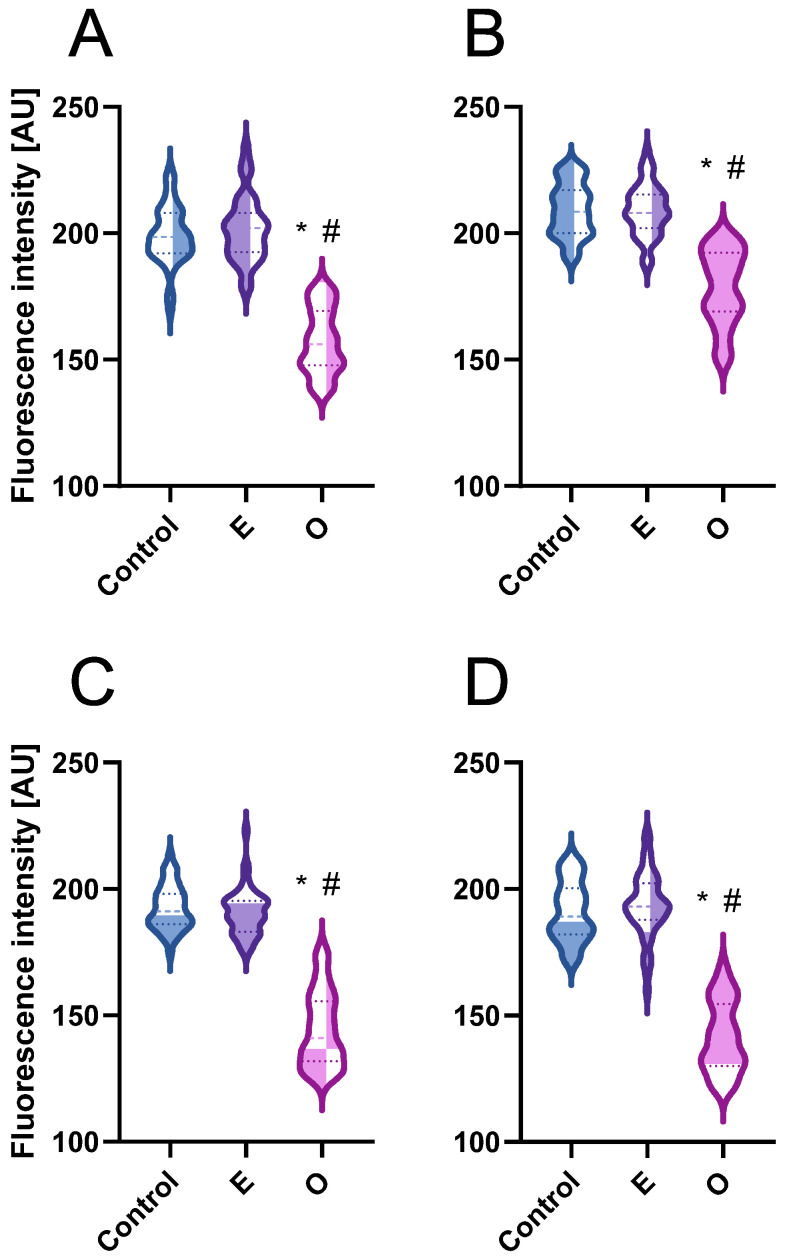
Mean fluorescence intensity of EdU-positive nuclei from root meristem cells in the S-phase [arbitrary units—AU] of *V. faba* (**A**), *L. luteus* (**B**), *B. napus* (**C**), and *A. thaliana* (**D**) after 24 h seedling incubation in water—Control, the emulsifier solution—E, or emulsified SEO at the IC_50_ concentration—O. The width of each violin plot represents the distribution of values along the y-axis; medians are indicated by dashed lines and quartiles by dotted lines. Statistical differences were assessed using the Mann–Whitney test at *p* ≤ 0.05. Asterisk (*) and hash (#) marks denote significant differences compared to the control and the emulsifier, respectively.

Fluorescence intensity measurements of replicating cells revealed that SEO not only reduced the number of replicating cells but also significantly limited the incorporation of the marker into newly synthesized DNA strands (Figure 6). This reduction, characterized by weaker fluorescence intensity on the nuclear surface compared to the control, was more pronounced in Brassicaceae (70% of the control) than in Fabaceae (approximately 80% of the control; see Figure 6A–D).

**Figure 7:**
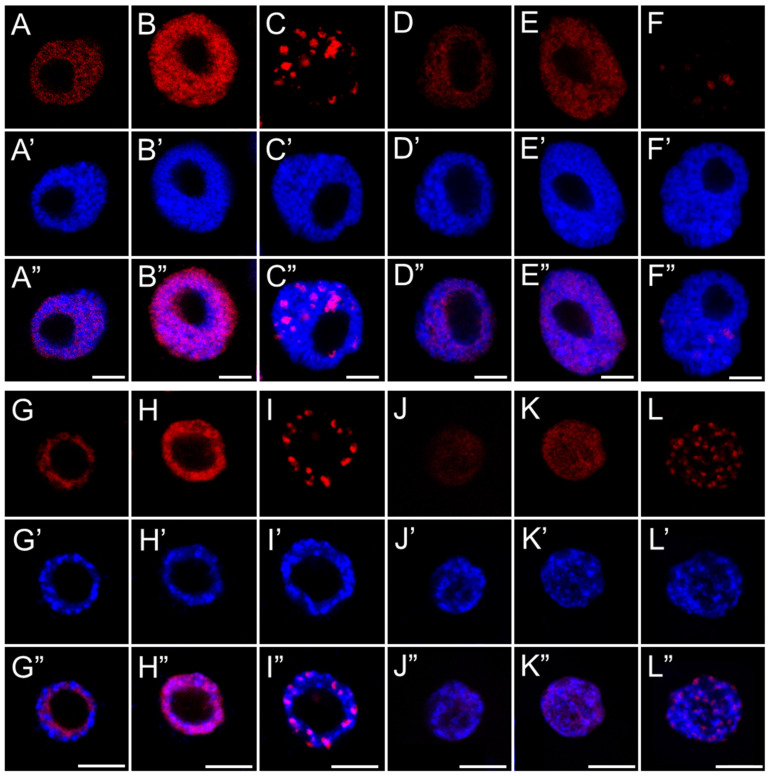
EdU-positive nuclei of root apical meristem cells of *V. faba* (**A**–**F**) and *L. luteus* (**G**–**L**) at three replication stages: early (**A**,**D**,**G**,**J**), mid (**B**,**E**,**H**,**K**), and late S-phase (**C**,**F**,**I**,**L**) after 24 h seedling incubation in water—Control (**A**–**C**,**G**–**I**), or emulsified SEO at IC_50_ concentration (**D**–**F**,**J**–**L**). Corresponding DNA staining with DAPI is shown in (**A’**–**L’**), and merged images in (**A”**–**L”**). Scale bar: 5 µm.

**Figure 8:**
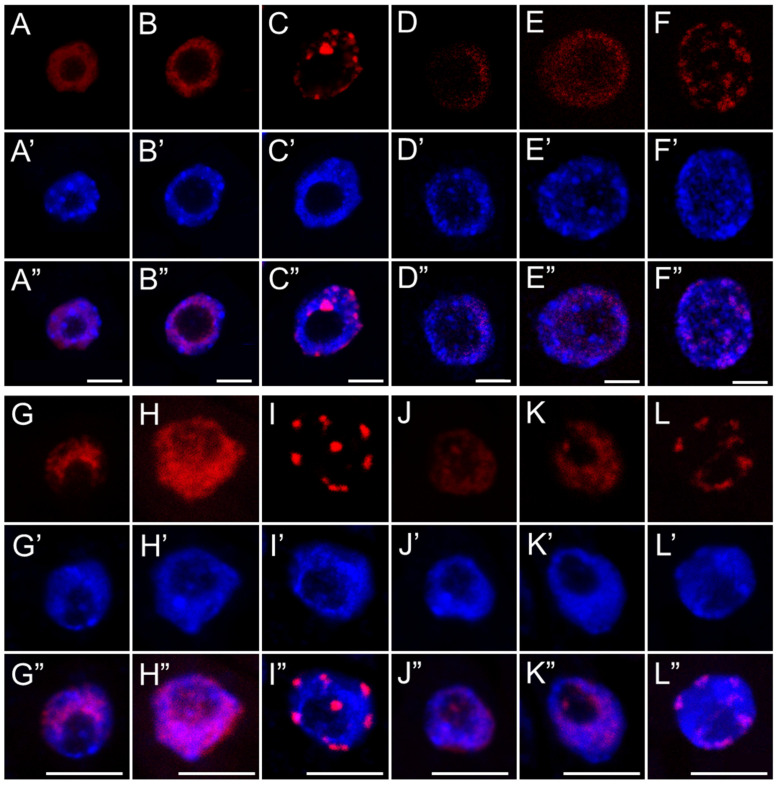
EdU-positive nuclei of root apical meristem cells of *B. napus* (**A**–**F**) and *A. thaliana* (**G**–**L**) at three replication stages: early (**A**,**D**,**G**,**J**), mid (**B**,**E**,**H**,**K**), and late S-phase (**C**,**F**,**I**,**L**) after 24 h seedling incubation in water—Control (**A**–**C**,**G**–**I**), or emulsified SEO at IC_50_ concentration (**D**–**F**,**J**–**L**). Corresponding DNA staining with DAPI is shown in (**A’**–**L’**), and merged images in (**A”**–**L”**). Scale bar: 5 µm.

**Figure 9:**
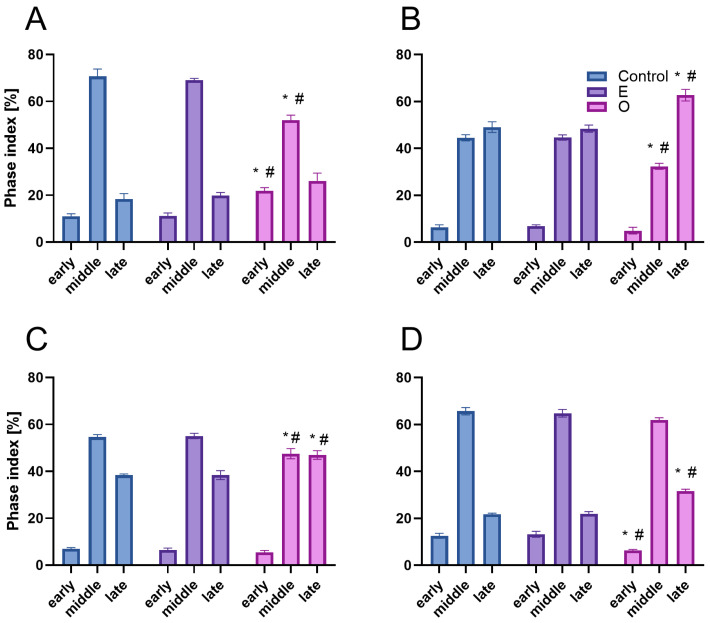
Fractions of nuclei [%] in root apical meristem cells of *V. faba* (**A**), *L. luteus* (**B**), *B. napus* (**C**), and *A. thaliana* (**D**) at early, mid, and late S-phase after 24 h seedling incubation in water—Control, the emulsifier solution—E, or emulsified SEO at the IC_50_ concentration—O. Data are presented as mean indices ± SEM from three biological replicates. Statistical differences were assessed using Student’s *t*-test at *p* ≤ 0.05. Asterisk (*) and hash (#) marks indicate significant differences compared to the control and the emulsifier, respectively.

The application of a replication marker also allowed us to distinguish nuclei at three different stages of DNA replication (Figure 7 and Figure 8) (1) an early stage. It is characterized by a small number of active replicons and, consequently, only a few fluorescent signals within the nucleus, which is similar in size to those in the G1 phase; (2) a middle stage, marked by the presence of numerous replicons and intense marker incorporation, visualized as a large number of homogeneously distributed foci occurring in nuclei that are noticeably larger than those in the early stage; and (3) a late stage, distinguished by fluorescent signals forming clusters that indicate late-replicating heterochromatin regions, in nuclei with a surface area comparable to those in the G2 phase. The analysis of the percentage distribution of nuclei at different stages of the S phase (Figure 9) clearly revealed an increase in the subpopulation of nuclei with a clustered labeling pattern, characteristic of heterochromatin replication, in the meristems of all SEO-treated species, at the expense of a decrease in the number of homogeneously labeled nuclei.

### 2.3. Evaluation of DNA Damage

**Figure 10:**
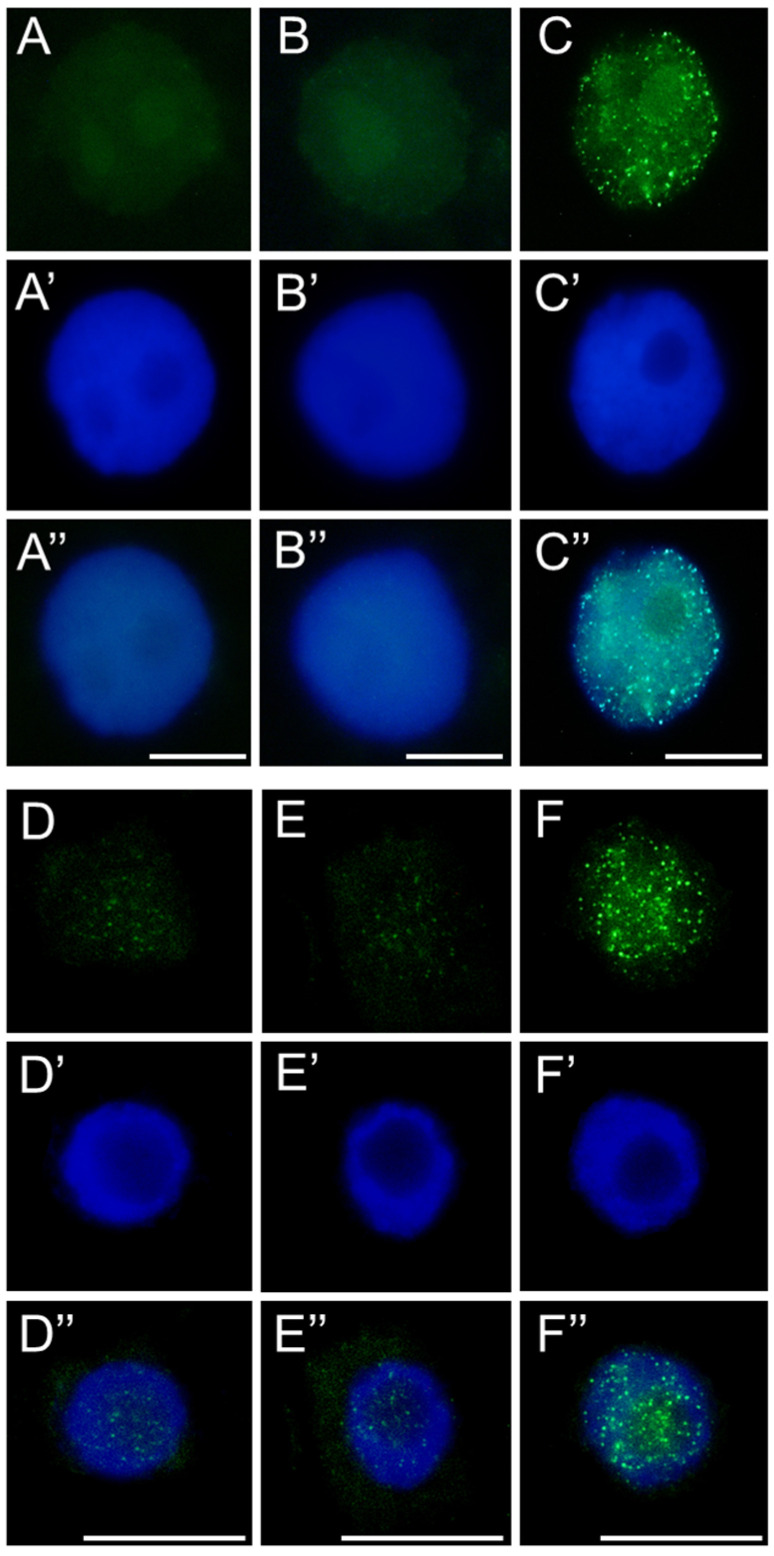
DNA breaks visualized by immunolabelling of γH2AX histone modification in root meristem cells of *V. faba* (**A**–**C**) and *B. napus* (**D**–**F**) after 24 h seedling incubation in water—Control (**A**,**D**), the emulsifier solution (**B**,**E**), or emulsified SEO at the IC_50_ concentration (**C**,**F**). Corresponding DNA staining with DAPI is shown in (**A’**–**F’**), and merged images in (**A”**–**F”**). Scale bar: 10 µm.

**Figure 11:**
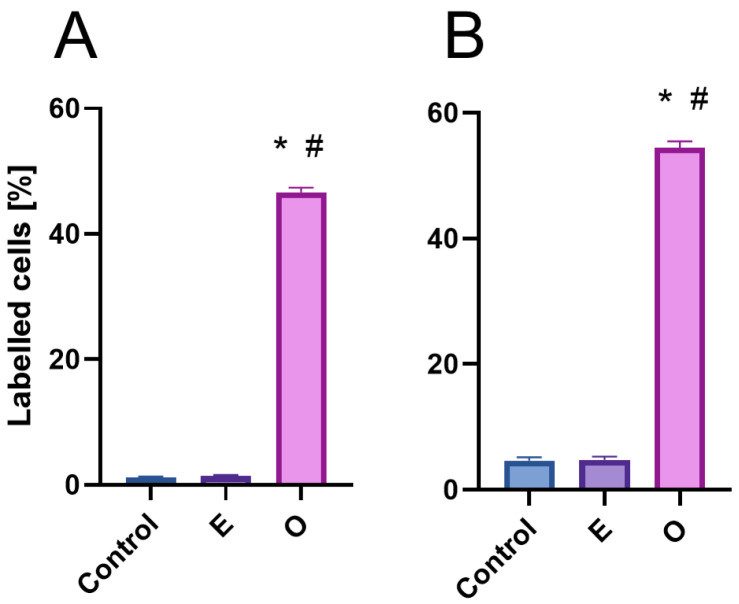
Frequencies of γ-H2AX positive nuclei [%] in root meristem cells of *V. faba* (**A**) and *B. napus* (**B**) after 24 h seedling incubation in water—Control, the emulsifier solution—E, or emulsified SEO at the IC_50_ concentration—O. Data are presented as mean indices ±SEM from three biological replicates. Statistical differences were assessed using Student’s *t*-test at *p* ≤ 0.05. Asterisk (*) and hash (#) marks indicate significant differences compared to the control and the emulsifier, respectively.

Disruptions in the DNA replication process in meristems exposed to SEO may have been caused by strand integrity damage induced by excessive levels of reactive oxygen species. Therefore, immunodetection of γ-H2AX histones, a specific modification of histone H2AX that localizes to sites of double-strand breaks (DSBs) in DNA, was performed. The study was conducted on representatives of the Fabaceae (*V. faba*) and Brassicaceae (*B. napus*) families (Figure 10). Fluorescence microscopy observations revealed a weak positive signal in a few meristem cells of both the control and emulsifier-treated seedlings, indicating naturally occurring breaks during DNA replication and repair (Figure 10A,B,D,E and Figure 11). However, after 24 h of SEO treatment, the number of labeled cells increased to approximately 50%, with clearly visible numerous fluorescence foci in the nuclear area confirming the presence of double-strand DNA breaks (Figure 10C,D and Figure 11).

**Figure 12:**
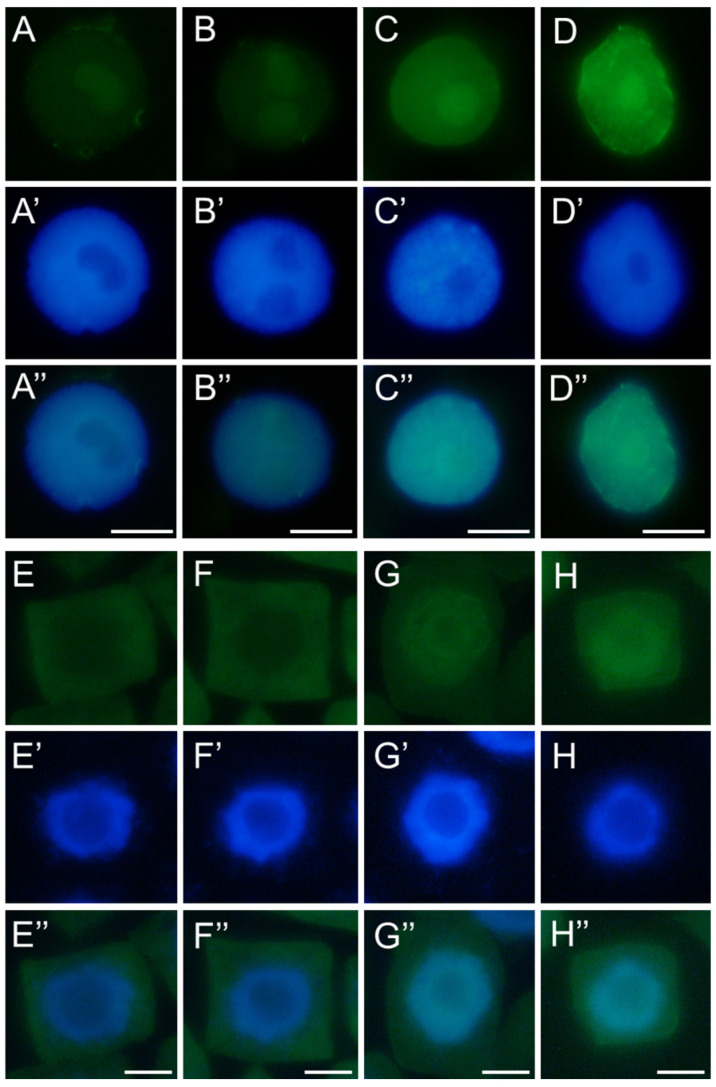
Detection of DNA breaks using the TUNEL assay in nuclei of root meristem cells of *V. faba* (**A**–**D**) and *B. napus* (**E**–**H**) after 24 h seedling incubation in water—Control (**A**,**E**), the emulsifier solution (**B**,**F**), or emulsified SEO at the IC_50_ concentration (**C**,**G**). Positive control (DNase I treatment; (**D**,**H**)). Corresponding DNA staining with DAPI is shown in (**A’**–**H’**), and merged images in (**A”**–**H”**). Scale bar: 10 µm.

**Figure 13:**
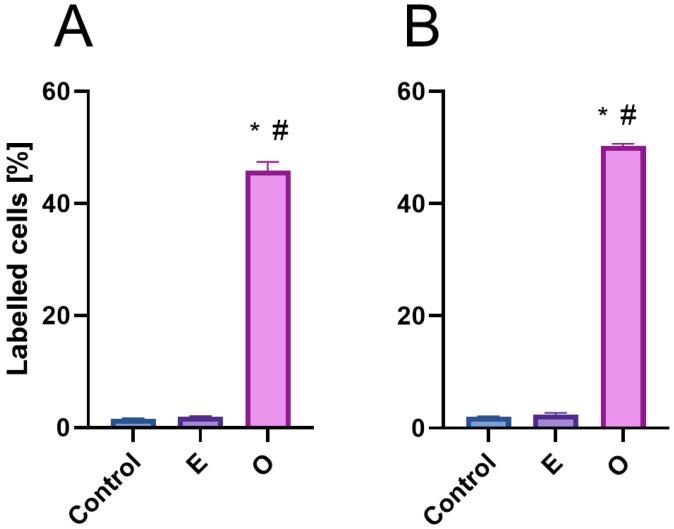
Frequencies of TUNEL-positive nuclei in root meristem cells of *V. faba* (**A**) and *B. napus* (**B**) after 24 h seedling incubation in water—Control, the emulsifier solution—E, or emulsified SEO at the IC_50_ concentration—O. Data are presented as mean indices ±SEM from three biological replicates. Statistical differences were assessed using Student’s *t*-test at *p* ≤ 0.05. Asterisk (*) and hash (#) marks indicate significant differences compared to the control and the emulsifier, respectively.

The TUNEL method (Terminal deoxynucleotidyl transferase dUTP Nick-End Labeling) detects both single-strand (SSB) and double-strand (DSB) DNA breaks, which may result from yet unrepaired genetic material damage as well as severe ones leading to cell death. Terminal deoxynucleotidyl transferase (TdT), a key enzyme in the assay, incorporates dUTP nucleotides into free 3′-OH DNA ends formed during fragmentation, allowing subsequent visualization of DNA nicks via the Click-iT reaction. Microscopic observations revealed dark, non-fluorescent nuclei in control cells and weak fluorescence in the nuclei of root meristematic cells treated with SEO (compare Figure 12A–C or Figure 12E–G). Analysis of labeling indexes showed that approximately 50% of cells were positively stained after 24 h IC_50_ SEO treatment in both tested species (Figure 13).

**Figure 14:**
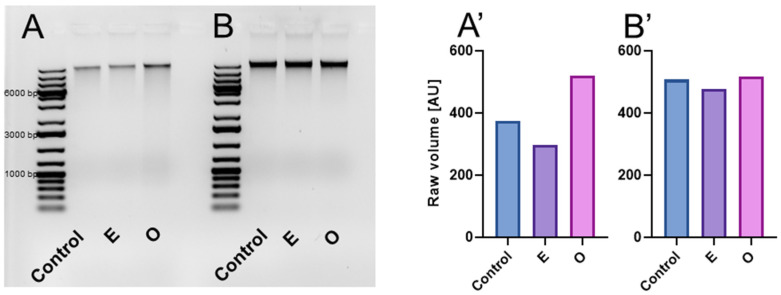
Gel electrophoresis of DNA (**A**,**B**) and analysis of smear volume [arbitrary units, AU] (**A’**,**B’**). DNA was isolated from apical parts of *V. faba* (**A**,**A’**) and *B. napus* (**B**,**B’**) roots excised from seedlings after 24 h incubation in water—Control, the emulsifier solution—E, or emulsified SEO at the IC_50_ concentration—O.

DNA gel electrophoresis is a widely used method for evaluating DNA damage by separating DNA fragments in an agarose gel, resulting in either a DNA ladder pattern (more characteristic of PCD) or a smear pattern (more characteristic of necrosis). Electrophoretic separation of total genomic DNA isolated from primary roots of *V. faba* and *B. napus* treated with water, emulsifier, and SEO did not reveal DNA fragmentation in the form of DNA laddering. However, a slight smear, indicating possible DNA damage, was observed only in SEO-treated *V. faba* seedlings (Figure 14).

## 3. Discussion

In response to the growing demand for environmentally safe and effective bioherbicidal substances, recent studies have increasingly focused on the phytotoxic effects of essential oils [5,21]. This progress has been made possible thanks to the development of modern research techniques, which allow for more precise identification of the cellular targets and affected biochemical mechanisms of action of these substances in plant cells [22,23,24,25]. As a result, these studies go beyond those assessing only the general effects of essential oils on seed germination or seedling growth [26,27]. However, these efforts remain insufficient, as essential oils are complex mixtures that are difficult to evaluate unambiguously, plant species exhibit varying degrees of sensitivity to their action, and many preliminary findings still require further confirmation and elaboration [28].

Our studies focus on evaluating the bioherbicidal activity of essential oil extracted from *Acorus calamus* rhizomes (SEO) [19] against two economically important crop species (*V. faba*, *L. luteus*) from the Fabaceae family and two weed species (*B. napus*, *A. thaliana*) from the Brassicaceae family. *V. faba* and *L. luteus* are among the most widely cultivated large-seeded legumes; however, their slow initial growth under natural conditions, combined with the limited availability of dedicated herbicides, promotes the aggressive spread of competing weeds [29,30]. Although *B. napus* is a crop species, its volunteer plants pose serious threats to legume cultivation [31]. Meanwhile, *A. thaliana* serves both as a weed and as a model organism in molecular studies. The selection of *Acorus calamus* essential oil for this study was motivated by its unique chemical composition, particularly the prevalence of phenylpropanoids such as asarones [19,26], which are known for their redox-modulating properties. Practical factors, including the oil’s economic viability, the easy accessibility of *A. calamus*, and its large rhizomes, also contributed to the choice. Despite its long-standing use in traditional medicine and demonstrated bioactivity in animal models and in vitro systems [27,32,33], the molecular mechanisms of its action in plant cells remain poorly understood.

The initial stage of the study demonstrated that an appropriately selected concentration of SEO can significantly inhibit the growth of the primary roots in Brassicaceae seedlings without disrupting primary root growth in Fabaceae, and even stimulating more intensive root development in *L. luteus* [20]. Based on the IC_50_ values determined for each species (*V. faba*—0.03%, *L. luteus*—0.025%, *B. napus*—0.01%, and *A. thaliana*—0.05%), it was possible to compare responses across taxonomic groups, investigate the cellular targets of SEO under equivalent stress intensity, and assess the potential for species- or family-specific sensitivity patterns. Analyses of oxidative stress levels and the antioxidant system response as well as general metabolic changes (via isothermal calorimetry) in primary roots confirmed the existence of distinct physiological strategies for coping with SEO exposure after 24 h incubation at IC_50_ concentrations [20].

Primary root growth depends on both cell elongation in the elongation zone and cell proliferation occurring in the root apical meristem. While our previous study focused on general physiological and metabolic responses to SEO at the whole-organ level, the present work investigates early molecular targets within the root meristem. The accumulation of reactive oxygen species (ROS) is one of the earliest indicators of oxidative stress and can significantly influence not only antioxidant system activity and cellular metabolism but also genome stability and cell cycle progression [13,23,34]. Therefore, in this study, we visualized and quantified ROS levels in meristematic cells exposed to SEO and analyzed the progression of DNA replication during the S phase. Although root growth in the analyzed species was inhibited by 50%, ROS levels in the meristems varied, even among species within the same family, suggesting differences in stress tolerance. Variations in ROS accumulation were observed for different Fabaceae species under *Eucalyptus saligna* leaf litter treatment [35], as well in *Brassica* species under drought stress [36], which confirms our assumption.

The stress markers detected in root meristem cells accumulated mainly in cytoplasmic organelles located near the nuclei, leaving the nuclear area noticeably lighter and free of pigment deposits. This is likely due to the fact that the nucleus is not a primary site of ROS production, and even if ROS or staining reagents such as DAB reach the nucleus, the absence of peroxidases, required to catalyze the chromogenic reaction, prevents visible pigment formation. In all analyzed species, oxidized DAB (indicating H_2_O_2_ accumulation) was primarily localized in large organelles previously identified as plastids by electron microscopy [37,38] whereas formazan (indicating the presence of O_2_^•−^) accumulated predominantly in much smaller organelles identified as mitochondria [38]. In non-photosynthetic tissues, mitochondria are the main source of O_2_^•−^, produced during the activity of the electron transport chain. The accumulated O_2_^•−^ is highly reactive and does not freely diffuse; instead, it is rapidly converted into H_2_O_2_ by superoxide dismutase (SOD) [39]. What is more, ROS generation by essential oil treatment could not be the only reason responsible for organelle damage. Molecular docking studies in the course of evaluating the *Artemisia argyi* essential oil against weed species revealed the oil components exhibit binding affinity with 4-hydroxyphenylpyruvate dioxygenase enzymes. These are responsible for plastid quinones biosynthesis, and their inhibition results in affected carotenoid biosynthesis and, in turn, chloroplast dysfunction reflected in leaf bleaching and decreased pigment levels [40].

Mitochondria and plastids are highly sensitive cellular sensors of environmental changes and function as signaling hubs, maintaining bidirectional communication with the cell nucleus, which modulates gene expression in response to intracellular disturbances [41,42,43]. Given the presence of organelle-to-nucleus (retrograde) signaling pathways, it is plausible that, despite the absence of visible ROS marker accumulation in the nucleus, appropriate oxidative stress signals generated in the organelles were relayed to the nucleus via specific signaling cascades [43]. One well-characterized mechanism in plants involves the transcription factor ANAC017, which, in response to organelle-derived stress, is released from the ER membrane and translocated to the nucleus, where it activates the expression of stress-responsive genes [44]. Additionally, both mitochondria and plastids are capable of releasing calcium ions (Ca^2+^) under oxidative stress. The resulting increase in cytosolic Ca^2+^ levels is detected and relayed by specific calcium-binding sensor proteins, such as calmodulin (CaM), calcineurin B-like proteins (CBLs), and calcium-dependent protein kinases (CDPKs), which initiate downstream signaling [45]. Furthermore, MAP kinases (e.g., MPK3 and MPK6) can phosphorylate proteins, including transcription factors, in response to stress signals and promote the activation of defense-related genes in the nucleus [14]. Together, these signaling pathways suggest a coordinated interplay among mitochondria, plastids, and the nucleus in orchestrating gene expression essential for cell survival during oxidative stress. Although our study did not directly investigate nuclear gene expression, ANAC017 translocation, or calcium signaling components, these well-established pathways provide a conceptual framework for interpreting the observed stress responses and merit further targeted investigation in future research on the cellular effects of SEO in plants.

Although our study showed that H_2_O_2_ predominantly accumulates in organelles, this relatively stable molecule is known to not only participate in signaling but also diffuse into the nucleus, where its excess may induce oxidative damage to DNA [46]. While we did not directly assess nuclear H_2_O_2_ levels, previous findings have demonstrated its ability to permeate cellular membranes—including the nuclear envelope—and to generate DNA lesions. These include oxidative base modifications such as guanine oxidation to 8-oxoG, single- and double-strand breaks, and the subsequent activation of the ATM/ATR–SOG1 signaling cascade, which regulates DNA repair and can induce cell cycle arrest at the G1/S checkpoint or during replication [17]. Alternatively, like other DNA-damaging factors, H_2_O_2_ could shift the cell cycle toward endoreplication when genome integrity is compromised [47,48,49]. However, our cytophotometric analysis of cell nuclei from all tested species, following 24 h seedling incubation with IC_50_ SEO concentrations did not reveal any nuclei with DNA content exceeding 4C. This suggests that the level or duration of stress was insufficient to trigger endocycle entry, or alternatively, that meristematic cells of these species do not typically engage in endoreplication under this type of oxidative challenge. Instead, we observed an increase in the population of nuclei in the G1 phase, pointing to the possible activation of the G1/S checkpoint. In plants, this control mechanism, referred to as Principal Control Point 1 (PCP1), integrates signals related to cellular metabolism, energy status (including ATP availability), nucleotide pools, and genomic integrity to determine whether replication should proceed [29,37,38].

Despite the substantial accumulation of reactive oxygen species (ROS) in mitochondria of meristematic root cells treated with SEO—potentially contributing to ATP depletion due to intensified mitochondrial electron transport activity [50]—the energy reserves were likely not sufficiently exhausted to fully activate PCP1. This conclusion is supported by the observation that, although reduced compared to control meristems, a significant proportion of cells still initiated DNA synthesis: approximately 70% of the control level in Fabaceae and 80% in Brassicaceae. Under conditions of severe energy or nutrient limitation, the cell cycle is typically arrested at PCP1, preventing EdU incorporation into nuclear DNA [51]. However, cells exposed to SEO exhibited notably weaker nuclear fluorescence signals from incorporated EdU compared to the control. This reduction may, in part, stem from limited EdU uptake. Prior to incorporation into DNA, EdU must be transported across the plasma membrane via nucleoside transporters and phosphorylated by nucleoside kinases [52]. Our earlier findings of increased lipid peroxidation under SEO treatment suggest impaired membrane integrity, potentially affecting nucleoside transport. Moreover, severe mitochondrial stress could hinder ATP production, further limiting EdU activation via phosphorylation. Nevertheless, since PCP1 did not block entry into S phase, the EdU signal likely reflects reduced replication fork progression and/or a limited number of simultaneously active replicons. This interpretation aligns with previous studies showing that volatile monoterpenes can slow down replication in the meristematic cells of *Brassica campestris* [53]. The observed effects may be linked to oxidative stress signals from mitochondria or plastids, possibly indicating damage to organellar DNA during interphase replication. Such disturbances could indirectly influence the rate of nuclear DNA synthesis [42,53]. Additionally, they may point to replication-associated obstacles within the nucleus itself that require time to resolve. This hypothesis is supported by the strong nuclear labeling of the modified histone variant H2AX (γ-H2AX), which marks sites of double-strand breaks (DSBs). The presence of γ-H2AX confirms that replication slowdown may result from S-phase checkpoints activation in response to DNA damage, most likely mediated by ATM/ATR kinases and the plant-specific transcription factor SOG1—a functional analog of p53 in animal cells—initiating the DNA repair response.

Although γ-H2AX accumulation might also suggest early stages of programmed cell death, it more likely reflects the mild genotoxic potential of SEO, which does not lead to extensive cellular degradation. Hallmarks of advanced cell death, such as the formation of apoptotic-like bodies or DNA laddering observed during electrophoretic DNA separation, were not detected, supporting this interpretation. These findings are consistent with results obtained for meristematic cells of *Lactuca sativa* L. treated with *Backhousia citriodora* L. essential oil, where DNA fragmentation was minimal, despite detectable lesions in the TUNEL assay and the absence of DNA laddering in electrophoretic analysis [54]. In contrast, seedlings of *Lactuca sativa* L. treated with juglone clear DNA showed degradation in the TUNEL assay, accompanied by a characteristic smear on DNA electrophoresis, indicative of extensive damage [55].

Despite the presence of DNA lesions, replication proceeds in SEO-treated cells, with nuclei in all analyzed species progressing to the final stage of DNA synthesis, during which heterochromatin, organized into the chromocenters, is replicated. Interestingly, the proportion of nuclei at this late replication stage (distinguished by clustered fluorescent signals) increased in SEO-treated meristems, which may point to difficulties in repairing DNA within highly condensed chromatin regions [56]. This suggests that the checkpoint system may retain cells at this stage, providing additional time for repair processes to be completed [12]. Nevertheless, further investigation is required to determine whether the accumulation of nuclei at this phase indeed results from checkpoint-mediated delays in response to unresolved replication-associated DNA damage within condensed chromatin regions.

The genotoxic effects observed in this study are likely attributable to the presence of asarones, the principal constituents of SEO [57]. Although no similar studies have examined the impact of *A. calamus* essential oil or its individual components in plant cells, their effects have been assessed in human liver carcinoma (HepG2) cells. In that study, HepG2 cells were incubated with EC_50_ concentrations of α- and β-asarone, as well as with asarone epoxides—metabolic derivatives of asarones. The results showed that α-asarone, and especially asarone epoxide, exerted stronger biological activity than β-asarone, which is already considered a potent genotoxic agent [58]. DNA strand breaks were detected as early as 1 h after incubation, accompanied by activation of DNA repair mechanisms, as indicated by increased ATM kinase expression 2 h after exposure to asarone epoxide. The findings suggest that the genotoxicity of asarones may stem from their metabolic conversion into reactive epoxides [58] and raise the question of whether similar metabolic activation mechanisms exist in plant cells.

Taken together, our results suggest that while treatment with SEO at IC_50_ concentration for 24 h induces a measurable genotoxic stress response, the damage remains within a threshold that permits the continuation of replication, albeit with delays and activation of S-phase checkpoints. However, preliminary experiments conducted during IC_50_ selection revealed that doubling the concentration to 2 × IC_50_ resulted in more severe nuclear alterations, including chromatin disorganization and impaired replication dynamics. These observations imply that prolonged exposure or higher doses may shift the cellular response toward irreversible damage. Further studies are planned to confirm this dose-dependent effect and to characterize the extent and nature of replication disturbances and genotoxic lesions.

## 4. Materials and Methods

### 4.1. Plant Material

Seeds of 4 plant species: faba bean (*Vicia faba* L. subsp. *minor*, cv. Bobas; Danko, HR Sp. z o. o. Choryń, Poland), yellow lupine (*Lupinus luteus* L., cv. Baryt; PHR Sp. z o. o. Tulce, Poland), rapeseed (*Brassica napus* L. cv. Markus; HR Strzelce Sp. z o. o. Strzelce, Poland), and thale cress (*Arabidopsis thaliana* Col-0 (L.) Heynh.; NASC, Nottingham, UK) were germinated on filter paper moistened with deionized water in Petri dishes for 2–3 days at 23 °C in darkness, except *A. thaliana*, which germinated in long-day conditions (16/8 h) due to etiolation sensitivity. Seedlings with equal-sized primary roots (about 2 cm *V. faba*, *L. luteus*, or 1 cm *B. napus* and 0.5 cm *A. thaliana*) were selected for further experiments. In addition to their agricultural importance, species selection was guided by technical criteria relevant to cytological and molecular studies. Among the Fabaceae, *Vicia faba* var. *minor* was chosen for its large genome size, clearly visible interphase nuclei with a reticulate structure and prominent chromocenters, as well as large, easily distinguishable chromosomes suitable for damage assessment. In contrast, *Lupinus luteus* has considerably lower DNA content and displays nuclei with a more dispersed chromatin structure, facilitating the detection of interphase-level damage. Within the Brassicaceae, *Brassica napus*, also characterized by low DNA content, displays a similar chromatin organization and nuclear type, and performs well in immunocytochemical analyses. *Arabidopsis thaliana* was included as a reference model species with a well-characterized genome, enabling future molecular analyses, including gene expression studies.

### 4.2. Essential Oil Treatment

For performing the treatment, sweet flag essential oil (SEO) (Etja, Elbląg, Poland) was used. To decrease the tension between the oil and water phases, the SEO in an emulsified form was prepared. The emulsifier (consisted of C14-18 and unsaturated C16-18—mono- and di-ethoxylated glycerides and ethoxylated *B. napus* oil) was mixed with SEO in a 1:4 (*v*/*v*) ratio and vigorously mixed by vortexing for 15 min. For seedling incubation, the emulsified SEO was diluted in water to concentrations corresponding to the IC_50_ values. To ensure a comparable stress level and standardize plant responses, IC_50_ values were determined individually for each tested species: 0.03% for *V. faba*, 0.025% for *L. luteus*, 0.01% for *B. napus*, and 0.005% for *A. thaliana* [20]. These concentrations represent the doses that inhibited embryonic root growth by 50%. This experimental setup enables a detailed investigation of SEO’s mode of action, allowing the identification of physiological and molecular responses under moderate stress conditions. Such conditions do not cause organismal death but instead trigger defense mechanisms and induce metabolic changes. Additionally, this approach facilitates comparisons of cellular behavior across species, helping to determine whether the observed responses are conserved, species-specific, or show patterns characteristic of particular plant families [22]. Seedlings of all four species were incubated on filter paper in tightly sealed with Parafilm Petri dishes, in darkness, at 23 °C for 24 h, using SEO concentrations corresponding to the IC_50_ values. Seedlings incubated in water and dissolved emulsifier in the concentration used for SEO emulsification were considered as control. Ten root meristems were examined per species (Control series, emulsifier series—E, and SEO series—O), and four experimental repeats were performed, resulting in a total of n = 40 root meristems per species per treatment.

### 4.3. DNA Staining for Cytophotometry

Excised 1 cm long root segments were fixed in Carnoy’s solution (99.8% ethanol: glacial acetic acid, 3:1 *v*/*v*) for 1 h at room temperature, rinsed in absolute ethanol (3 times), and stored in 70% ethanol. Before DNA staining, root tips were rehydrated, hydrolyzed in 4M HCl (for 2h *V. faba*; 1h *L. luteus*, *B. napus*; and 30 min *A. thaliana*) and stained with Schiff’s reagent (pararosaniline; Sigma-Aldrich, St. Louis, MI, USA) for 1 h using the standard Feulgen method [59]. Root meristems were cut off, squashed in a drop of 45% acetic acid onto microscopic slides (Menzel-Gläser, Thermo Fisher Scientific, Waltham, MA, USA), and placed on the surface of a dry ice cube. Following freezing with dry ice, coverslips were removed, and the preparations were rinsed in 70% ethanol, air-dried, and covered in a drop of Canada balsam.

### 4.4. Detection of a Replication Process

The Click-iT^®^ EdU Alexa Fluor^®^ 555 Imaging Kit (Thermo Fisher Scientific, Waltham, MA, USA) was used to visualize DNA synthesis. Seedlings were incubated on a watch glass in 10 µM 5-ethynyl-2′-deoxyuridine (EdU) solution for 30 min, at room temperature in darkness. Then, roots were fixed in 0.01 M PBS-buffered 4% paraformaldehyde solution (4 °C; pH 7.4) for 40 min and rinsed twice in 0.01 M PBS buffer. Next, root tips were macerated with a 0.1 M citrate-buffered mixture of 2.5% pectinase, 2.5% pectolyase, and 2.5% cellulase (Sigma Aldrich) at pH 5.0 and 37 °C for 30 min (*V. faba* and *L. luteus*), 20 min (*B. napus)*, or 15 min (*A. thaliana*). Rinsed twice in PBS, meristems were squashed in a drop of 0.01 M PBS onto Super Frost Plus microscope slides (Menzel-Gläser). Following freezing with dry ice, coverslips were removed, and slides were air-dried. After washing with 0.01 M PBS, DNA replication in nuclei was visualized by the Click-iT reaction carried out according to the manufacturer’s protocol. After the reaction, slides were washed with 0.01 M PBS, stained with 15 µM 4′,6-diamidino-2-phenylindole (DAPI, Sigma-Aldrich) for 15 min, rewashed with 0.01 M PBS, and mounted in 0.01 M PBS/glycerol/DABCO (2.3% diazabicyclo [2.2.2]octane) mixture.

### 4.5. Detection of Apoptosis-like Programmed Cell Death (Al-PCD)

Due to the comparable replication patterns observed across all four species and technical limitations of immunocytochemical analyses, two representative species—one from Fabaceae and one from Brassicaceae—were selected for further investigation. Root meristems of *V. faba* and *B. napus* were fixed in 0.01 M PBS-buffered 4% paraformaldehyde for 40 min at 4 °C, rinsed twice in 0.01 M PBS buffer, and macerated with a 0.1 M citrate-buffered enzyme mixture of 2.5% pectinase, 2.5% pectolyase, and 2.5% cellulase (Sigma Aldrich) at 37 °C for 30 min (*V. faba*) or 20 min (*B. napus*). A TUNEL assay was performed according to the manufacturer’s instruction (Click-iT^®^ TUNEL Alexa Fluor^®^ 488 Imaging Assay (Thermo Fisher Scientific). Cell nuclei were stained with DAPI (15 µM 4′,6-diamidino-2-phenylindole; Sigma-Aldrich) for 15 min and rinsed again with 0.01 M PBS (3 times for 10 min each). Microscopic slides were mounted in 0.01 M PBS/glycerol/DABCO mixture.

DNA was isolated from root tips using NucleoSpin Plant II, Mini kit for DNA from plants (Macherey-Nagel) according to the manufacturer’s instructions. DNA samples (2 µg DNA each) were separated in agarose gel electrophoresis (1%) in a 1X Tris-acetate-EDTA (TAE) buffer containing ethidium bromide (1 mg ∙ mL^−1^) at 100 V for 3 h at RT. Gel was visualized using ProXima 2750 imaging platform (Isogen Life Sciences, De Meern, The Netherlands).

### 4.6. Immunocytochemical Staining of γ-Phosphorylated H2AX Histones

The same two representative species were used for this part of the study, as justified in the previous section. Apical parts of *V. faba* and *B. napus* roots, fixed in 0.01 M PBS-buffered 4% paraformaldehyde (4 °C, 40 min) and macerated with a 0.1 M citrate-buffered enzyme mixture of 2.5% pectinase, 2.5% pectolyase, and 2.5% cellulase (Sigma Aldrich) at 37 °C for 30 min (*V. faba*) or 20 min (*B. napus*), were squashed onto Super Frost Plus microscope slides (Menzel-Gläser) and frozen on a dry ice cube (as described above in the procedure for identifying the replication process). Then air-dried samples were pretreated with 0.01 M PBS-buffered 5% bovine serum albumin (BSA, Sigma Aldrich) and 4% Triton X-100 (Sigma Aldrich) at room temperature for 50 min, and subsequently incubated overnight at 4 °C in darkness and a humidified atmosphere with primary rabbit polyclonal antibodies against human γ-H2AX histones phosphorylated at Ser139 (Thermo Fisher Scientific), diluted 1:750 in antibody buffer (1% BSA; 0.3% Triton X-100 in 0.01 M PBS). In the next day, slides were rinsed with 0.01 M PBS (3 times for 10 min) and incubated at 23 °C in darkness for 90 min with the secondary mouse, anti-rabbit antibodies, conjugated with Alexa Fluor^®^ 488 dissolved in 1:600 ratio in antibody buffer. After rinsing with 0.01 M PBS (3 times for 10 min each), cell nuclei were stained with DAPI (15 µM 4′,6-diamidino-2-phenylindole; Sigma-Aldrich) for 15 min and rinsed again with PBS (3 times for 10 min each). After that, the slides were embedded in 0.01 M PBS/glycerol mixture (9:1) with 2.3% DABCO.

### 4.7. H_2_O_2_ and O_2_^•−^ Detection in Root Meristematic Cells

For H_2_O_2_ detection, seedlings of *V. faba*, *L. luteus*, *B. napus*, and *A. thaliana* after 24 h incubation in water, emulsifier, and IC_50_ emulsified SEO were incubated for 1 h at room temperature in 1 mg ∙ mL^−1^ diaminobenzidine tetrachloride solution (DAB-HCl; Sigma-Aldrich) in 0.01 M PBS buffer and rinsed twice in 0.01 M PBS buffer. Then, excised root tips were fixed in 4% paraformaldehyde buffered with 0.01 M PBS for 40 min at 4 °C, rinsed twice in 0.01 M PBS, and macerated with a 0.1 M citrate-buffered enzyme mixture (2.5% pectinase, 2.5% pectolyase and 2.5% cellulase; Sigma Aldrich) at pH 5.0 and 37 °C for 30 min (*V. faba* and *L. luteus*), 20 min (*B. napus)*, or 15 min (*A. thaliana*). The samples were then washed and embedded in 0.01 M PBS/glycerol mixture (9:1) with 2.3% DABCO.

To visualize O_2_^•−^ seedlings of *V. faba* and *B. napus* after 24 h incubation in water, emulsifier, and IC_50_ emulsified SEO were incubated for 10 min at room temperature in a 3 mM 0.01 M PBS-buffered nitroblue tetrazolium (NBT; Sigma Aldrich) and rinsed twice in 0.01 M PBS buffer after the incubation stopped. The next steps considering slide preparation were similar to those for DAB staining.

### 4.8. Microscopic Observations, Measurements, and Analyses

DNA content was evaluated with a Jenamed 2 microscope (Carl Zeiss, Jena, Germany). The extinction of Feulgen-stained cell nuclei was measured at 565 nm and calibrated in arbitrary units, taking the values recorded for half-telophases and prophases from control roots as reference standards of 2C and 4C DNA levels, respectively. A computer program (Forel, Łódź, Poland) was used for image analysis using micro densitometry to evaluate DNA content from 5000 nuclei from five meristems.

Z-stacks of EdU-treated cells were acquired using a 0.3 µm step size under oil immersion using Leica Laser Scanning Confocal Microscopy (LSCM) SP8 platform (Leica Microsystems, Wetzlar, Germany) using 100×/1.40 OIL lens, with the use of Laser Line Supercontinuum Visible (555 nm) and UV (405 nm) Diode laser and visualized using Leica LAS X software ver. 2.0.2.15022. Proportions [%] of early-, mid-, and late-S-phase cells in meristems of *V. faba* seedlings, calculated by combining microfluorimetric quantitation of DNA contents in DAPI-stained cell nuclei and visual EdU fluorescence analysis.

Observations and analyses of DAB, NBT, TUNEL and anti-γ-phosphorylated H2AX histone visualized cells were made using Nikon Eclipse E600W fluorescence microscope (Nikon, Tokyo, Japan) equipped with U2 filter (UVB light; λ = 340–380 nm) for DAPI, B2 filter (blue light; λ = 465–496 nm) for Alexa Fluor^®^ 488, and G2 filter (green light; λ = 540/25 nm) for Alexa Fluor^®^ 555. All images were recorded at the same time of integration using a DS-Fil CCD camera (Nikon). Quantitative color or fluorescence intensity analyses were made in ImageJ software ver. 1.54p [60] after converting color images into greyscale and expressed in arbitrary units.

Analysis of DNA gel electrophoresis was performed using GelAnalyzer 23.1.1 (available at www.gelanalyzer.com) by Istvan Lazar Jr., PhD and Istvan Lazar Sr., PhD. After loading the gel image, lanes and bands were automatically detected by the program. The smear volumes were measured by adjusting the band visible on the lane profile, namely, the threshold covering the band visible as a peak on the lane plot has been moved below the line and stopped at the nearest plateau. The smear volume was expressed in the Analysis info window in the Raw volume column.

### 4.9. Statistical Analyses

Statistical analyses and outcomes visualization were performed in GraphPad Prism 10 software. Differences between groups were evaluated using Student’s *t*-test or Mann–Whitney test. A *p*-value ≤ 0.05 was considered statistically significant and marked with asterisks (*) or hash (#).

## 5. Conclusions

**Figure 15:**
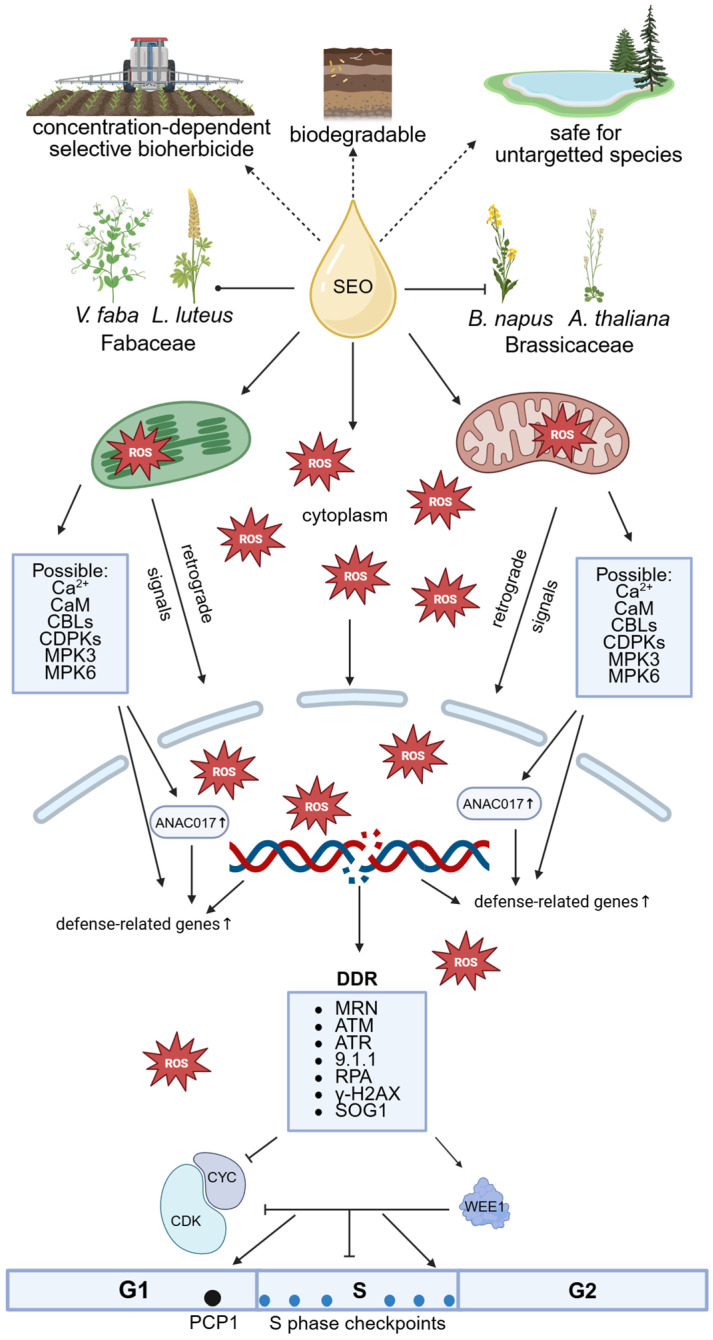
Proposed mechanism of action of *Acorus calamus* essential oil (SEO) at IC_50_ concentration in root meristematic cells of Fabaceae and Brassicaceae species. SEO induces organelle-specific oxidative stress, leading to DNA damage, activation of cell cycle checkpoints, and altered replication dynamics without triggering extensive cell death. These effects underpin its potential as a selective, biodegradable, and environmentally safe bioherbicide for sustainable weed management. Depending on concentration, SEO exhibits species-specific selectivity, enabling targeted suppression of Brassicaceae weeds in Fabaceae-dominated cropping systems (explanation of abbreviations in the work test). Created with Biorender.com.

Our study shows that meristematic cells of primary roots from four species—*Vicia faba*, *Lupinus luteus*, *Brassica napus*, and *Arabidopsis thaliana*, representing Fabaceae and Brassicaceae—exhibit a common response to SEO at its IC_50_ concentration. This includes oxidative stress, with ROS primarily accumulating in mitochondria and plastids, but also affecting the nucleus (Figure 15).

Oxidative stress, along with the direct action of SEO components, causes DNA double-strand breaks that trigger checkpoint activation and repair mechanisms, delaying cell cycle progression. As a result, more cells accumulate in G_1_ phase, DNA replication slows due to fewer active replicons or slower fork progression, and heterochromatin replication is impaired due to chromatin compaction. Despite evident genotoxicity, the absence of apoptotic-like fragmentation or endoreduplication suggests that SEO does not induce extensive cell death or trigger genome duplication. Instead, oxidative stress disrupts S phase progression, with species differing in severity, not in response type. Given the importance of accurate DNA replication for mitosis, our ongoing research investigates whether this replication stress impacts mitotic structures or chromosome behavior.

These findings support the further development of *Acorus calamus* essential oil (SEO) as a sustainable weed management tool. Future studies should first apply multi-omics approaches to clarify molecular mechanisms, followed by expanded species screening under laboratory and field conditions, and the optimization of application parameters and formulations.

Taken together, our results support the use of SEO as effective, selective, biodegradable, and environmentally responsible weed control tools, enabling targeted suppression of weeds or invasive plants while sparing crops and helping to address herbicide resistance. Such approaches hold promise for regulatory approval and societal acceptance, while potentially reducing chemical inputs, lowering production costs, and enhancing the long-term sustainability and resilience of agroecosystems.

## Data Availability

Data will be made available on request.

## Abbreviations

The following abbreviations are used in this manuscript:

9-1-1 Complex	RAD9-RAD1-HUS1 Complex
ATM	Ataxia-Telangiectasia Mutated Kinase
ATR	Ataxia-Telangiectasia and Rad3-Related Kinase
BSA	Bovine Serum Albumin
Ca^2+^	Calcium ions
CaM	Calmodulin
CBL	Calcineurin B-Like Protein
CDK	Cyclin-Dependent Kinase
CDPK	Calcium-Dependent Protein Kinase
CKI	Cyclin-Dependent Kinase Inhibitor
Cyc	Cyclin
DAB	3,3′-Diaminobenzidine
DABCO	2.3% diazabicyclo [2.2.2]octane;
DAPI	4′,6-Diamidino-2-Phenylindole
DDR	DNA Damage Response
EdU	5-Ethynyl-2′-Deoxyuridine
H_2_O_2_	Hydrogen Peroxide
IC_50_	Half-inhibitory Concentration
KRP	Kip-Related Protein
MPK	Mitogen-Activated Protein Kinase
MRN	MRE11-RAD50-NBS1 Complex
NBT	Nitro blue tetrazolium chloride
O_2_^●−^	Superoxide Anion Radical
PBS	Phosphate-Buffered Saline
AL-PCD	Apoptosis-Like Programmed Cell Death
PCP1	Principal Control Point 1
Rb	Retinoblastoma Protein
ROS	Reactive Oxygen Species
RPA	Replication Protein A
SEO	*Acorus calamus* essential oil
SOG1	Suppressor of Gamma Response 1
TUNEL	Terminal Deoxynucleotidyl Transferase dUTP Nick-End Labeling
γ-H2AX	Phosphorylated Histone H2AX
